# MiR-590-5P Inhibits Growth of HepG2 Cells via Decrease of S100A10 Expression and Inhibition of the Wnt Pathway

**DOI:** 10.3390/ijms14048556

**Published:** 2013-04-18

**Authors:** Xiangxiang Shan, Yufeng Miao, Rengen Fan, Haixin Qian, Ping Chen, Hongqi Liu, Xiaomei Yan, Jianping Li, Fen Zhou

**Affiliations:** 1 Department of Geraeology, Yancheng City No.1 People’s Hospital, Yancheng 224001, China; E-Mail: fanrengen2@sina.com; 2 Department of General Surgery, the First Affiliated Hospital of Soochow University, Suzhou 215006, China; E-Mails: miaoyufeng823@sohu.com (Y.M.); lanyieryuan@sohu.com (H.Q.); 3 Department of Medical Oncology, Yancheng City No.1 People’s Hospital, Yancheng 224001, China; E-Mails: ccpping@163.com (P.C.); liuhongqi@163.com (H.L.); anxiaomei@163.com (X.Y.); lijp79@163.com (J.L.); zzzfffen@163.com (F.Z.); 4 Department of General Surgery, Yancheng City No.1 People’s Hospital, Yancheng 224001, China; E-Mail: rengenf@163.com

**Keywords:** miR-590-5P, S100A10, hepatocellular carcinoma, Wnt pathway, lentiviral system, reporter gene

## Abstract

Hepatocellular carcinoma is one of the most common and lethal cancers worldwide, especially in developing countries. In the present study, we found that the expression of a microRNA, miR-590-5P, was down-regulated and S100A10 was up-regulated in six hepatocellular carcinoma cell lines. The reporter gene assay showed that overexpression of miR-590-5P effectively reduced the activity of luciferase expressed by a vector bearing the 3′ untranslated region of S100A10 mRNA. Ectopic miR-590-5P overexpression mediated by lentiviral infection decreased expression of S100A10. Infection of Lv-miR-590-5P inhibited cell growth and induced cell cycle G1 arrest in HepG2 cells. In addition, miR-590-5P expression suppressed the expression of Wnt5a, cMyc and cyclin D1, and increased the phosphorylation of β-catenin and expression of Caspase 3, which may contribute to the inhibitory effect of miR-590-5P on cell growth. Taken together, our data suggest that down-regulation of miR-590-5P is involved in hepatocellular carcinoma and the restoration of miR-590-5P can impair the growth of cancer cells, suggesting that miR-590-5P may be a potential target molecule for the therapy of hepatocellular carcinoma.

## 1. Introduction

Hepatocellular carcinoma (HCC) is one of the most common cancers worldwide, characterized by high incidence, difficulty to cure and high mortality. It is the second major cause of cancer mortality in China, accounting for 53% of total deaths caused by HCC in the world [1]. The increasing incidence and mortality rates result in a severe burden on patients, their families and society. The generation and development of HCC is a complex process in which a great variety of factors and steps are involved, and the mechanisms have not been clearly elucidated until now. Identifying the genes mediating this process and understanding the molecular genetics of HCC is urgent, in order to provide a theoretical basis for diagnosis and treatment.

S100A10 (also known as Annexin II light chain or p11) belongs to the S100 protein family, a highly conserved group of low-molecular weight EF-hand calcium-binding proteins. The mutations in its two EF-hands eliminate its calcium binding capacity [2]. Usually, S100A10 exists in cells together with its natural ligand, annexin A2 (p36), in the annexin A2 heterotetramer (AIIt) [3–7]. Annexin A2 sub-unit mediates the membrane localization of the AIIt complex. The interaction between S100A10 and a variety of receptors (e.g., 5-HTR1B and 5-HTR4) and channel proteins (e.g., TASK-1, TRPV5 and TRPV6) is shown to mediate their cell surface presentation and functional expression [8–12]. S100A10 can also bind to plasminogen and induces plasminogen activation by interaction with plasminogen activators through its *C*-terminal lysine residues, facilitating the conversion of plasminogen to plasmin [13]. Further genetic studies have shown that S100A10 plays a crucial role in the recruitment of macrophages to the tumor site, which may be mediated by S100A10-stimulated plasmin generation [14]. We have analyzed S100A10 expression in hepatocellular carcinoma and paraneoplastic tissues from clinical samples and the results show that S100A10 contents were increased in cancer tissues. Therefore, further investigation is required to reveal its role in hepatocellular carcinoma and the regulatory mechanism.

MicroRNAs (miRNAs) are a kind of endogenous short non-coding RNAs of ~22 nucleotides naturally occurring in many eukaryote organisms, including plants, *C. elegans*, Drosophila and mammals [15,16]. MiRNAs usually bind to partially complementary sites within the 3′ untranslated region (3′-UTR) of their target mRNAs and consequently lead to translational blockage or mRNA degradation, so as to negatively regulate gene expression [17,18]. Each miRNA regulates scores of target genes, and an individual mRNA may be regulated by multiple miRNAs, giving rise to an extensive regulatory networks. MiRNAs play crucial roles in a diverse array of physiological and pathological processes, such as embryogenesis, development, cell differentiation, cell proliferation, cell death, tumorigenesis, as well as circadian rhythms [19–23]. Besides, it has been well established that miRNAs mediate several disease-related processes, including carcinogenesis. Recent studies have shown that known oncogenic miRNAs are up regulated in the liver of rats exposed to tamoxifen for 24 weeks [24]. Previously, Meng F *et al*. demonstrated that abnormal expression of miR-21 contributes to HCC growth and spreads through PTEN-dependent pathways [25]. In the present study, we identified another miRNA, miR-590-5P, which is involved in the growth and cell cycle regulation of hepatocellular carcinoma cells and explored implicated mechanisms through molecular and cellular techniques.

## 2. Results and Discussion

### 2.1. MiR-590-5P Is Down-Regulated and S100A10 Is Enhanced in Liver Cancer Cell Lines

**Figure 1:**
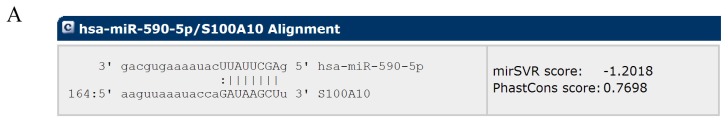

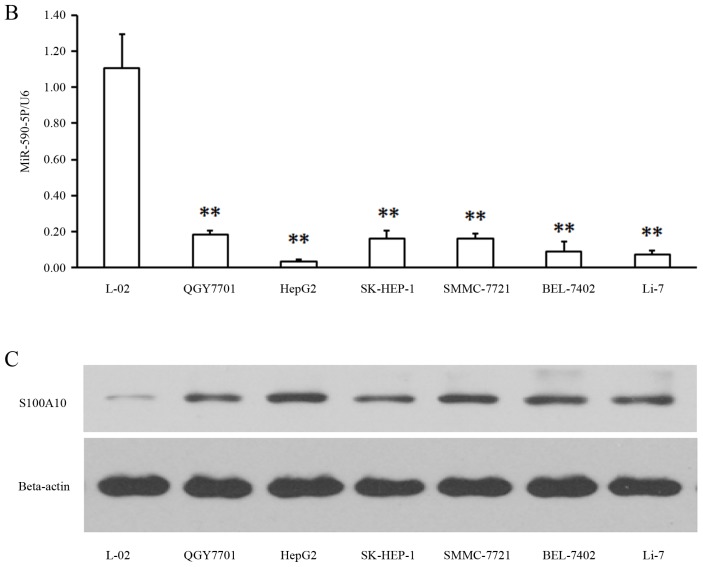
Bioinformatic analysis on the sequences of miR-590-5P and 3′-UTR of S100A10 and expression levels of miR-590-5P and S100A10 in human hepatocarcinoma cell lines and a normal liver cell line. (**A**) Alignment between miR-590-5p and S100A10 from microRNA.org (Targets and Expression); (**B**) Total RNA was extracted from the cells, and reversely transcribed and analyzed by quantitative PCR, and the expression level of miR-590-5P was normalized with internal control U6. The results are given as mean ± SD of three independent experiments; (**C**) Cell lysates of the indicated cells were prepared, and S100A10 levels were determined by specific primary antibody against S100A10 followed by HRP-linked secondary antibody. The bands were visualized by enhanced a chemiluminescence detection system. One representative of three independent experiments is shown.

Real time PCR and western blotting were used to detect miR-590-5P and S100A10 levels in various human hepatocarcinoma cell lines and a normal liver cell line, respectively. Expression of miR-590-5P was decreased significantly in all human hepatocarcinoma cells lines detected (*p* < 0.01, Figure 1B) in comparison with L-02 cells. Conversely, S100A10 protein contents in hepatocarcinoma cell lines were higher than those in normal liver cells (Figure 1C), compared to L-02 cells. The results that miR-590-5P expression was down-regulated in human hepatocarcinoma cells and S100A10 was highly expressed in hepatocarcinoma cells together with the bioinformatic analysis on the sequences of miR-590-5P and 3′-UTR of S100A10 (Figure 1A) suggest there may be a negative correlation between them both. Hence, we used the luciferase reporter system to further investigate the regulatory role of miR-590-5P in the expression of S100A10.

### 2.2. MiR-590-5P Inhibits S100A10 Expression by Interacting with 3′UTR of S100A10 mRNA

**Figure 2:**
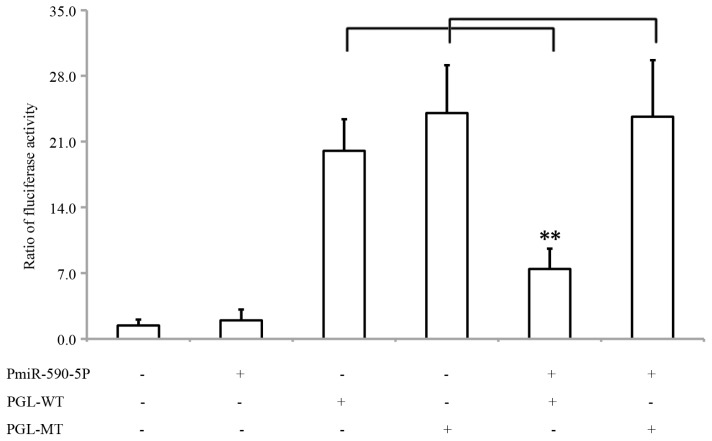
The 3′UTR of S100A10 enables miR-590-5P regulation. 293 cells were transfected with pmiR-590-5P construct with or without pGL-WT or pGL-MT. The histogram indicates relative firefly luciferase activities in the different transfected groups. Error bars represent standard deviation and were obtained from three independent experiments **, *t*-test significant at *p* < 0.01.

**Figure 3:**
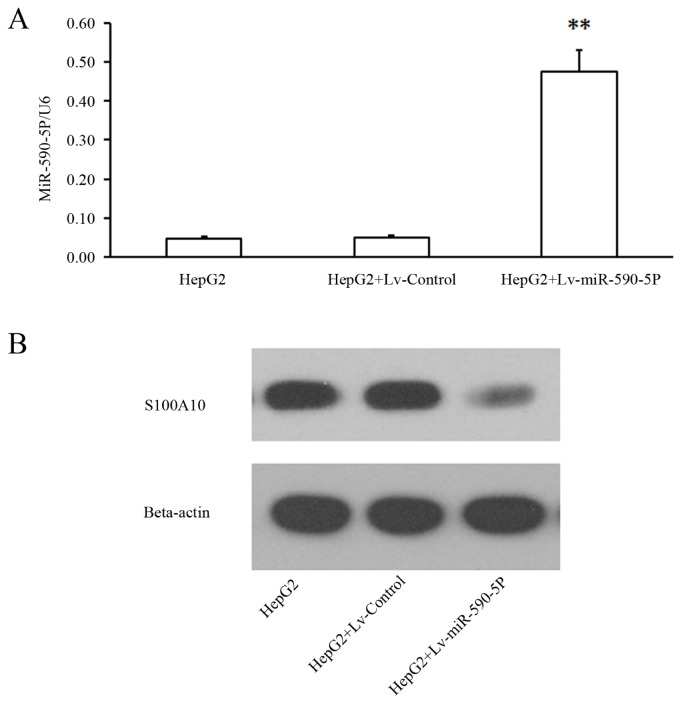
S100A10 is down-regulated by infection of Lv-miR-590-5P. (**A**) HepG2 cells were infected with Lv-control or Lv-miR-590-5P. After 96 h, the cells were collected and total RNA was extracted and subjected to quantitative PCR. U6 was used as an internal standard; (**B**) HepG2 cells were infected with Lv-control or Lv-miR-590-5P, and after 96 hours, the cells were collected and total protein was extracted and subjected to western blotting. For western blotting, cell lysates were separated on a 14% (S100A10) or 13% (beta-actin) SDS polyacrylamidegel, and the blots were detected with a specific anti-S100A10 antibody, with beta-actin as an internal standard **, *t*-test significant at *p* < 0.01. Data were confirmed in triplicate experiments.

In order to confirm whether miR-590-5P inhibits the expression of S100A10 via the 3′UTR of S100A10 mRNA, we cloned a 568-bp genomic segment into a pcDNA-copGFP vector to construct a pmiR-590-5P vector which was designed to express exogenous miR-590-5P. The expression miR-590-5P in 293 cells transfected with pmiR-590-5P significantly reduced the activity of firefly luciferase expressed by pGL-WT, which contains a wild type 3′UTR of S100A10 mRNA (*p* < 0.01, Figure 2). In addition, the mutation of the binding site abolished the inhibitory effect of miR-590-5P on the expression of the luciferase reporter (*p* > 0.05, Figure 2). Moreover, we used pmiR-590-5P to produce a lentivirus, Lv-miR-590-5p, to infect HepG2 cells. Fluorescence microscopic results showed that infection efficiency of Lv-miR-590-5p was over 90% (data not shown), real time PCR showed the lentiviral system effectively expressed miR-590-5p in HepG2 cells and western results showed miR-590-5P expression suppressed S100A10 expression significantly (Figure 3).

### 2.3. The Over-Expression of miR-590-5P Inhibits Cellular Proliferation and Causes Cell Cycle Arrest in HepG2

**Figure 4:**
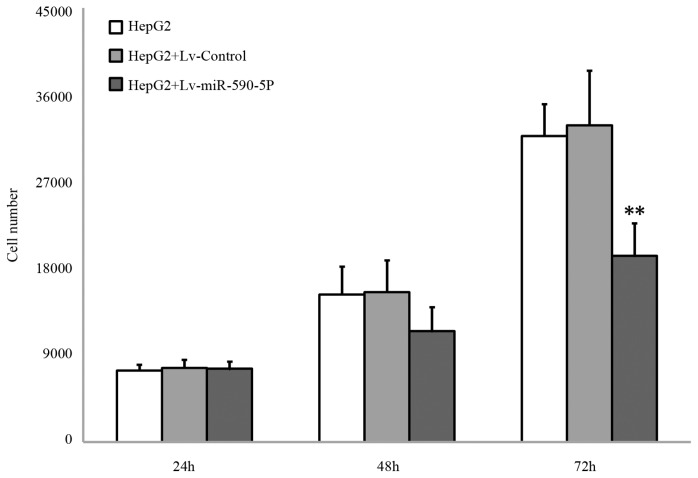
Effect of Lv-miR-590-5P on cell growth of HepG2 cells. HepG2 cells were infected by Lv-miR-590-5P or Lv-Control. The infected cells were reseeded to 96-wells plates. After incubation for indicated hours, the number of cells were detected by CCK-8 assay. The values represent the means ± SD (*n* = 3).

HEPG2 cells infected with Lv-miR-590-5P or Lv-control were subjected to proliferation assay. The results shown in Figure 4 suggest that exogenous expression of miR-590-5P inhibited the proliferation of HepG2 cells. After 24 h or 48 h incubation, HepG2 infected with Lv-miR-590-5P showed no remarkable morphological change compared to the control, and there was no difference in cell numbers between the two groups (*p* > 0.05) (Figure 4). However, the infection significantly inhibited the growth of HepG2 cells after 72 h, compared to the control group (*p* > 0.01) (Figure 4). The results suggested that miR-590-5P overexpression induces growth inhibition of HepG2 cells in a time-dependent manner.

**Figure 5:**
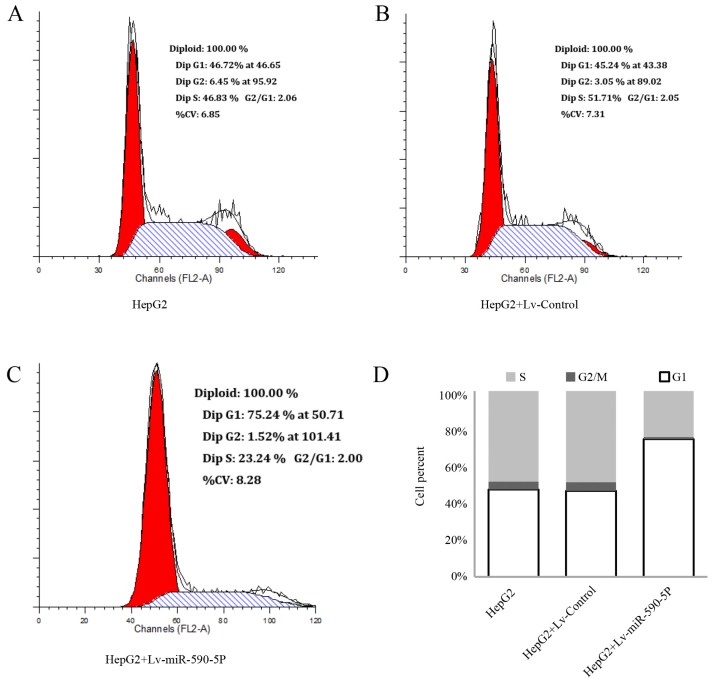
Effect of miR-590-5P on cell cycle in HepG2 cell line. (**A**), (**B**) and (**C**) Cell cycle analysis produced by flow cytometry in HepG2 cells which were infected with or without Lv-Control or Lv-miR-590-5P; (**D**) Cell cycle distribution in percentages of the different groups. Data were obtained by flow cytometry and presented as mean (*n* = 3).

To investigate whether the inhibition of miR-590-5P on cell proliferation of HepG2 was mediated by cell cycle alteration, cell cycle distribution was analyzed. Histograms of flow cytometric data are shown in Figure 5. The results show that in comparison with the control group, miR-590-5P expression significantly increased cell population at the G1 phase (Figure 5), from 46.92% ± 4.17% to 74.09% ± 6.09% (*p* > 0.01) (Figure 5D), indicating that overexpression of miR-590-5P causes a cell cycle G1 arrest in HepG2 cells.

### 2.4. The Over-Expression of miR-590-5P Affects Wnt Pathway Signaling in HepG2 Cells

**Figure 6:**
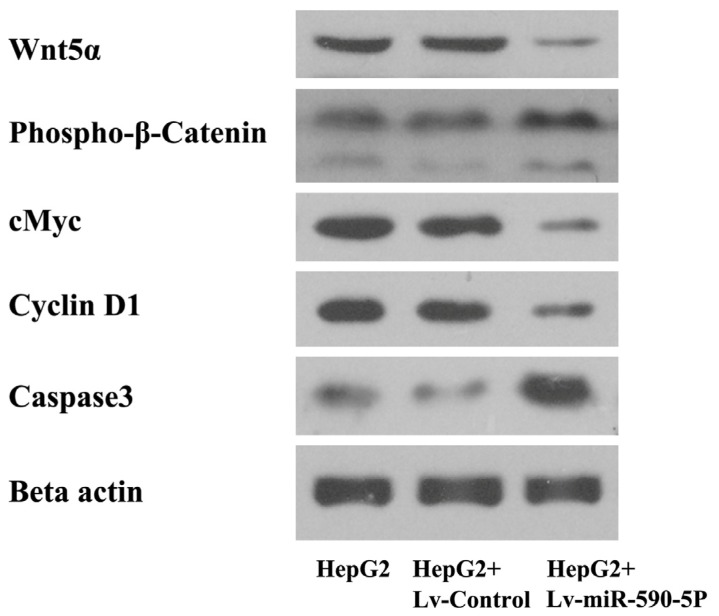
Effects of miR-590-5P on the Wnt pathway and downstream proteins. HepG2 cells were infected with or without Lv-Control or Lv-miR-590-5P. Ninety six hours later, expression of Wnt5a, cMyc, cyclin D1, Caspase 3 and phosphorylation levels of β-catenin were determined by western blotting, using β-actin as a loading control.

Since Wnt signaling pathway is an important pathway involved in primary liver cancer, we then detected several proteins and downstream target proteins in Wnt signaling pathway by western blotting. Results showed that lentiviral-mediated over-expression of miR-590-5P reduced the expression of Wnt5a and increased the phosphorylation of β-catenin (Figure 6). A protooncogene downstream of the Wnt singling pathway, cMyc, was decreased by miR-590-5P over-expression. A similar result was obtained for cyclin D1, an important regulator for cell cycle progression. Conversely, Caspase 3 was increased in HepG2 by the over-expression of miR-590-5P.

Since miRNAs are involved in many diverse biological processes and pathological processes, they have become one of the maine focuses in basic research and the research and development of applications [26]. By regulating genes through partially complementary sequences, miRNAs can influence extensively target genes. Recently, an increasing number of miRNAs were identified as playing roles in the carcinogenic process as oncogenes or as tumour suppressors, such as miR-335, miR-206, and miR-126 in breast cancer metastasis, miR-126, miR-143, and miR-145 in cervical cancer, and miRNA-451 in non-small cell lung cancer [27–29]. A previous study showed that, miR-590-5P can inhibit the metastasis of breast cancer [30]. In addition, it was found that miR-590-5P is down-regulated in cervical cancer samples [31]. All the above-mentioned evidence indicates that miR-590-5P may act as a potential tumor suppressor gene. Among all the studies on hepatocellular carcinoma, we have demonstrated for the first time that interaction between S100A10 and miR-590-5P is involved in hepatocellular carcinoma.

Firstly, it was found that the expression of miR-590-5P was down-regulated in a number of hepatocellular carcinoma cell lines. The down-regulation of miR-590-5P may result in the dysregulation of its target genes. Here, the luciferase reporter gene assay revealed that S100A10 is a target gene of miR-590-5P, and the mutation in the binding site at 3′-UTR of S100A10 mRNA eliminates the regulatory effect of miR-590-5P.

Secondly, a lentiviral vector was used to express miR-590-5P in HepG2 cells, since the lentiviral system has several significant advantages in comparison with other viral or non-viral gene delivery system, including extensive target cells, high-efficiency infection of dividing and non-dividing cells, the ability to contain a large exogenous sequence for tissue-specific expression, persistent expression by gene integration into genomic DNA and low immunogenicity and toxic response [32–35]. In this study, the lentivirus was used to evaluate the effects of miR-590-5P restoration on cell growth and cell cycle distribution. It was demonstrated that the lentiviral-mediated miR-590-5P expression efficiently reduced S100A10 protein and inhibited cell growth in HepG2 cells. Next, we also found that miR-590-5P restoration caused G1 phase arrest in HepG2 cells.

The Wnt gene family, of which the first member was identified as an oncogene involved in mouse breast cancers in 1982, plays a crucial role in the normal growth and development of mouse embryo [36]. Moreover, abnormal change in the Wnt signaling pathway, mediated by dysregulation or mutation of any component in the pathway, including β-catenin, adenomatous polyposis coli (APC) protein and glycogen synthase kinase 3β (GSK-3β), is found in a variety of cancers, including esophageal cancer, colorectal cancer, melanoma, and primary liver cancer [37–39]. Therefore, we detected the effects of miR-590-5P on the Wnt pathway and several down-stream proteins. The results showed that miR-590-5P expression decreased the expression of Wnt5a and increased the phosphorylation level of β-catenin, which can induce its degradation by the ubiquitin-proteasome system [40]. An oncogene, c-myc, was reduced as well, contributing to the inhibited proliferative activity of HepG2. Cyclin D1 was decreased by miR-590-5P. As an important regulator of G1 to *S*-phase transition, the decrease of cycling D1 may mediate G1 arrest of HepG2 cells. In addition, Caspase 3 was increased by over-expression of miR-590-5P, indicating that miR-590-5P may also enhance the apoptosis under the experimental conditions. All these changes may be responsible for the inhibitory effect of miR-590-5P on the growth of HepG2, though further studies are required to confirm the mechanisms.

## 3. Experimental Section

### 3.1. Cell Culture

L-02, QGY7701, HepG2, SMMC-7721, BEL-7402, Li-7, SK-HEP-1 and 293 cells were purchased from Cell Resource Center, Shanghai Institutes for Biologic Sciences, Chinese Academy of Sciences. L-02, QGY7701, HepG2, SMMC-7721, BEL-7402, SK-HEP-1 and Li-7 cells were maintained in RPMI 1640 containing 10% fetal bovine serum (FBS) (Invitrogen, Carlsbad, CA, USA) in a humidified incubator with 5% CO_2_ at 37 °C and SK-HEP-1 cells were maintained in Eagle’s Minimum Essential Medium (EMEM) (Invitrogen) supplemented with 10% FBS under the same environment. 293 cells were cultured in Dulbecco’s Modified Eagle Medium (DMEM) (Invitrogen) plus 10% FBS.

### 3.2. Plasmid Construction

Human genomic DNA was extracted from L-02 cells and used for amplification of the template of the precursor sequence of miR-590-5P. The primers used were: 5′-GGAATTCTTCAGTTGTAA CCCAG-3′ and 5′-CGGGATCCTTGAGATGTCACCAA-3′. The PCR product was digested using EcoR I and BamH I and ligated into linear pCDH-EF1-GFP vector (System Biosciences) and transformed into DH5α competent cells. The obtained vector was called pmiR-590-5P vector. 3′-untranslated region (3′-UTR) of human S100A10 was amplified from cDNA obtained through the reverse transcription of total RNA of L-02 cells. The following primers were used: wild type: 5′-GCTCTAGAAATGAGCAGTTCGCTCCTCC-3′ and 5′-GCTCTAGAACAATACAAAAATC AAAAGCTTATC-3′; mutated type: 5′-GCTCTAGAAATGAGCAGTTCGCTCCTCC-3′ and 5′-GCTCTAGAAACAATACAAAAATCAAATATGATCCTGGTA-3′. The products were digested with Xba I and inserted into pGL3-promotor vector (Promega, Madison, WI, USA). The resulting vectors were called as pGL-WT and pGL-MT, respectively. All the sequences of constructed vectors were verified by PCR-agarose electrophoresis and sequence analysis.

### 3.3. Cell Transfection and Luciferase Assay

Using Lipofectamine 2000 according to the manufacturer’s instructions 293 cells were transfected with the pmiR-590-5P, pGL-WT and pGL-MT. Forty eight hours after transient transfection, the cells were harvested and luciferase assays were performed. The relative luciferase activities (ratios of firefly and renilla luciferase activity) of lysates were measured by the dual luciferase reporter assay system (Promega, Madison, WI, USA).

### 3.4. Lentivirus Package and Cell Infection

In DMEM supplemented with 10% FBS 293TN Producer Cell Line (SBI) were cultured. One day before transfection, cells were seeded at 5 × 10^5^ per 10 cm dish. Lenti-miR-590-5P vector and pPACK Packaging Plasmid Mix (SBI, Mountain View, CA, USA) were co-transfected using Lipofectamine 2000 (Invitrogen). After 24 h, the medium was replaced with DMEM plus 2% FBS. After 48 h, the supernatant was harvested, cleared by centrifugation at 5000 rpm at room temperature for 5 min, and passed through a 0.45 μm PVDF membrane. The viral titer was measured by gradient dilution according to the expression level of GFP following the manufacturer’s instructions. The packaged lentiviruses were named Lv-miR-590-5P and Lv-Control.

One day after seeding, HepG2 cells were infected with Lv-miR-590-5P or Lv-Control diluted by DMEM at a multiplicity of infection (MOI) of 10 and the medium was refreshed after 24 h; the infection efficiency was assessed by fluorescence microscopy 96 h after infection. The expression of miR-590-5P in the infected HepG2 cells was detected by quantitative PCR. Then, the cells were subject to western blot analysis of S100A10, cell proliferation assay and other assays.

### 3.5. Real Time PCR

Total RNA was isolated from cells at the exponential phase or from HepG2 cells at 96 h after the infection of Lv-control or Lv-miR-590-5P using Trizol Reagent (Invitrogen) and was reversely transcribed into cDNA using M-MLV Reverse Transcriptase (Takara BIO, Dalian, China) with specific primers for U6 snRNA and has-miR-590-5p: 5′-TACCTTGCGAAGTGCTTAAAC-3′ and 5′-GTCGTATCCAGTGCGTGTCGTGGAGTCGGCAATTGCACTGGATACGACCTGCA-3′, respectively. Real-time PCR was performed using SYBR^®^ Premix Ex Taq™ kit (Takara, Dalian, China) and TP800 System (Takara). The following primers were used for quantification of human U6 snRNA and has-miR-590-5p: U6 snRNA: 5′-GTGCTCGCTTCGGCAGCACAT-3′ and 5′-TACCTTGCGAAGTGCTTAAAC-3′; and has-miR-590-5p: 5′-GAGCTTATTCATAAAAGT-3′ and 5′-TCCACGACACGCACTGGATACGAC-3′; 2 μL cDNA from the reverse transcription reaction was used as the template for real-time PCR. Cycling parameters were as follows: 40 cycles of denaturation at 95 °C for 10 s, annealing at 55 °C (U6) or 60 °C (miR-590-5p) for 20 s and extension at 72 °C for 20 s. The has-miR-590-5p levels were normalized to U6 using the delta-delta Ct method. Each RNA sample was run in triplicate.

### 3.6. Western Blotting

Western blotting was performed to detect the expression of S100A10 in several HCC cell lines and a normal liver cell line, as well as HepG2 cells infected with Lv-miR-590-5P or Lv-Control. After being cultured for 48 h, or 96 h after infection, the cells were harvested and total protein was isolated with M-PER® Mammalian protein extraction reagent (Pierce, Rockford, IL, USA) according to the manufacturer’s protocol. The protein concentrations were assessed using the BCA protein assay. Samples with the same amount of total protein were separated by SDS-PAGE using 14% (for S100A10) or 13% (for beta-actin) gel and transferred onto PVDF membranes (Millipore, Boston, MA, USA). The membranes were blocked with TBST containing 5% non-fat milk and incubated with antibodies against human S100A10 (1:200) (Santacruz) and beta-actin (1:1000) (Cell Signaling Technology) at 4 °C overnight. After washing, the membranes were then incubated with a HRP-conjugated anti-rabbit IgG antibody (Cell Signaling Technology, Boston, MA, USA) and detected by enhanced chemiluminescence reagents (Pierce). Beta-actin was used as an endogenous reference.

The change of expression of proteins involved into the WNT pathway and phosphorylation level of β-catenin in HepG2 cells infected by Lv-miR-590-5P were also assessed by western blotting. The Lv-miR-590-5P or Lv-control infected cells were harvested for protein extraction 96 h after infection. The antibodies against Wnt5a (1:1000) (Abcam, Cambridge, MA, USA), phospho-β-catenin (1:1000) (Abcam), cMyc (1:800) (Abcam), cyclin D1 (1:800) (Abcam) and beta-actin (1:800) (Cell Signaling Technology, Boston, MA, USA) were used to detected the corresponding proteins according to the instructions.

### 3.7. Cell Proliferation Assays

The control and miR-590-5P over-expression HepG2 cells were reseeded into 96-well plates at 1 × 104 cells per well. Then 10 μL CCK-8 solution (Dojindo, Kumamoto-ken, Japan) was added into each well at 24, 48 or 72 h after seeding. After incubation for an additional 4 h, the supernatants were transferred to new 96-well plates and the absorbance of each well at 450 m was determined.

Cell cycle analysis. HepG2 cells were infected with Lv-miR-590-5P or Lv-Control as described above. The cells at the logarithmic growth phase were typsinized, washed with phosphate buffered saline (PBS) twice, and then fixed with 70% ethanol at 4 °C overnight. The fixed cells were washed with PBS twice, resuspended in 100 μL PBS (containing 100 μg/mL ribonuclease A and 50 μg/mL propidium idide), and incubated at room temperature for 30 min. The cell cycle distribution was detected by a BD FACSCalibur flow cytometer.

### 3.8. Statistical Analysis

All values are presented as mean ± standard deviation (SD). Statistical significance was evaluated using the Student’s *t*-test for unpaired comparison; *p* values ≤ 0.05 were taken as the level of significance.

## 4. Conclusions

To sum up, this is the first study to demonstrate that lentivirus mediated over-expression of miR-590-5P in HepG2 cells efficiently inhibits S100A10 protein expression and cellular proliferation *in vitro*. Further detailed *in vitro* and *in vivo* studies should be carried out to provide more relevant information and hints for therapy development for human lung cancers.

## References

[1] Chen J.G., Zhang S.W. (2011). Liver cancer epidemic in China: Past, present and future. Semin. Cancer Biol.

[2] Santamaria-Kisiel L., Shaw G.S. (2011). Identification of regions responsible for the open conformation of S100A10 using chimaeric S100A11-S100A10 proteins. Biochem. J.

[3] Glenney J.R., Tack BF (1985). Amino-terminal sequence of p36 and associated p10: Identification of the site of tyrosine phosphorylation and homology with S-100. Proc. Natl. Acad. Sci. USA.

[4] Gerke V., Weber K. (1984). Identity of p36K phosphorylated upon Rous sarcoma virus transformation with a protein purified from brush borders; calcium-dependent binding to non-erythroid spectrin and F-actin. EMBO J.

[5] Waisman D.M., Annexin II (1995). tetramer: Structure and function. Mol. Cell. Biochem..

[6] Gerke V., Moss S.E. (1997). Annexins and membrane dynamics. Biochim. Biophys. Acta.

[7] Gerke V., Weber K. (1985). The regulatory chain in the p36-kd substrate complex of viral tyrosine-specific protein kinases is related in sequence to the S-100 protein of glial cells. EMBO J.

[8] Svenningsson P., Chergui K., Rachleff I., Flajolet M., Zhang X., El Yacoubi M., Vaugeois J.M., Nomikos G.G., Greengard P. (2006). Alterations in 5-HT1B receptor function by p11 in depression-like states. Science.

[9] Warner-Schmidt J.L., Flajolet M., Maller A., Chen E.Y., Qi H., Svenningsson P., Greengard P. (2009). Role of p11 in cellular and behavioral effects of 5-HT4 receptor stimulation. J. Neurosci.

[10] Girard C., Tinel N., Terrenoire C., Romey G., Lazdunski M., Borsotto M. (2002). p11, an annexin II subunit, an auxiliary protein associated with the background K^+^ channel, TASK-1. EMBO J.

[11] Renigunta V., Yuan H., Zuzarte M., Rinne S., Koch A., Wischmeyer E., Gao Y., Karschin A., Jacob R., Schwappach B. (2006). The retention factor p11 confers an endoplasmic reticulum-localization signal to the potassium channel TASK-1. Traffic.

[12] Van de Graaf S.F.J., Hoenderop J.G.J., Gkika D., Lamers D., Prenen J., Rescher U., Gerke V., Staub O., Nilius B., Bindels R.J. (2003). Functional expression of the epithelial Ca^2+^ channels (TRPV5 and TRPV6) requires association of the S100A10 annexin 2 complex. EMBO J.

[13] MacLeod T.J., Kwon M., Filipenko N.R., Waisman D.M. (2003). Phospholipid-associated annexin A2- S100A10 heterotetramer and its subunits: Characterization of the interaction with tissue plasminogen activator, plasminogen, and plasmin. J. Biol. Chem.

[14] Phipps K.D., Surette A.P., O’Connell P.A., Waisman D.M. (2011). Plasminogen receptor S100A10 is essential for the migration of tumor-promoting macrophages into tumor sites. Cancer Res.

[15] Bartel D.P. (2004). MicroRNAs: Genomics, biogenesis, mechanism, and function. Cell.

[16] He L., Hannon G.J. (2004). MicroRNAs: Small RNAs with a big role in gene regulation. Nat. Rev. Genet.

[17] Lewis B.P., Burge C.B., Bartel D.P. (2005). Conserved seed pairing, often flanked by adenosines, indicates that thousands of human genes are microRNA targets. Cell.

[18] Farh K.K., Grimson A., Jan C., Lewis B.P., Johnston W.K., Lim L.P., Burge C.B., Bartel D.P. (2005). The widespread impact of mammalian MicroRNAs on mRNA repression and evolution. Science.

[19] Pauli A., Rinn J.L., Schier A.F. (2011). Non-coding RNAs as regulators of embryogenesis. Nat. Rev. Genet.

[20] Leaman D., Chen P.Y., Fak J., Yalcin A., Pearce M., Unnerstall U., Marks D.S., Sander C., Tuschl T., Gaul U. (2005). Antisense-mediated depletion reveals essential and specific functions of microRNAs in Drosophila development. Cell.

[21] Shivdasani R.A. (2006). MicroRNAs: Regulators of gene expression and cell differentiation. Blood.

[22] Hwang H.W., Mendell J.T. (2006). MicroRNAs in cell proliferation, cell death, and tumorigenesis. Br. J. Cancer.

[23] Alvarez-Saavedra M., Antoun G., Yanagiya A., Oliva-Hernandez R., Cornejo-Palma D., Perez-Iratxeta C., Sonenberg N., Cheng H.Y. (2011). miRNA-132 orchestrates chromatin remodeling and translational control of the circadian clock. Hum. Mol. Genet.

[24] Pogribny I.P., Tryndyak V.P., Boyko A., Rodriguez-Juarez R., Beland F.A., Kovalchuk O. (2007). Induction of microRNAome deregulation in rat liver by long-term tamoxifen exposure. Mutat. Res.

[25] Meng F., Henson R., Wehbe-Janek H., Ghoshal K., Jacob S.T., Patel T. (2007). MicroRNA-21 regulates expression of the PTEN tumor suppressor gene in human hepatocellular cancer. Gastroenterology.

[26] Heneghan H.M., Miller N., Kerin M.J. (2010). MiRNAs as biomarkers and therapeutic targets in cancer. Curr. Opin. Pharmacol.

[27] Negrini M., Calin G.A. (2008). Breast cancer metastasis: A microRNA story. Breast Cancer Res.

[28] Wang X., Tang S., Le S.Y., Lu R., Rader J.S., Meyers C., Zheng Z.M. (2008). Aberrant expression of oncogenic and tumor-suppressive microRNAs in cervical cancer is required for cancer cell growth. PLoS One.

[29] Wang X.C., Tian L.L., Jiang X.Y., Wang Y.Y., Li D.G., She Y., Chang J.H., Meng A.M. (2011). The expression and function of miRNA-451 in non-small cell lung cancer. Cancer Lett.

[30] Singh P., Soon P.S., Feige J.J., Chabre O., Zhao J.T., Cherradi N., Lalli E., Sidhu S.B. (2012). Dysregulation of microRNAs in adrenocortical tumors. Mol. Cell. Endocrinol.

[31] Zheng Z.M., Wang X. (2011). Regulation of cellular miRNA expression by human papillomaviruses. Biochim. Biophys. Acta.

[32] Pfeifer A., Ikawa M., Dayn Y., Verma I.M. (2002). Transgenesis by lentiviral vectors, Lack of gene silencing in mammalian embryonic stem cells and preimp lantation embryos. Proc. Natl. Acad. Sci. USA.

[33] Yu X., Zhan X., D’Costa J., Tanavde V.M., Ye Z., Peng T., Malehorn M.T., Yang X., Civin C.I., Cheng L. (2003). Lentiviral vectors with two independent internal promoters transfer high-level expression of multiple transgenes to human hematopoietic stem-progenitor cells. Mol. Ther.

[34] Lois C., Hong E.J., Pease S., Brown E.J., Baltimore D. (2002). Germline transmission and tissue-specific expression of transgenes delivered by lentiviral vectors. Science.

[35] Lai Z., Brady R.O. (2002). Gene transfer into the central nervous system *in vivo* using a recombinant lentivirus vector. J. Neurosci. Res.

[36] Noda T., Oki S., Kitajima K., Harada T., Komune S., Meno C. (2012). Restriction of Wnt signaling in the dorsal otocyst determines semicircular canal formation in the mouse embryo. Dev. Biol.

[37] Veeck J., Dahl E. (2012). Targeting the Wnt pathway in cancer: The emerging role of Dickkopf-3. Biochim. Biophys. Acta.

[38] Camilli T.C., Weeraratna A.T. (2010). Striking the target in Wnt-y conditions: Intervening in Wnt signaling during cancer progression. Biochem. Pharmacol.

[39] Myant K., Sansom O.J. (2011). Wnt/Myc interactions in intestinal cancer: Partners in crime. Exp. Cell. Res.

[40] Daniel J.M., Reynolds A.B. (1997). Tyrosine phosphorylation and cadherin/catenin function. Bioessays.

